# Growth Hormone Promotes Hepatic Triglyceride Export in Humans

**DOI:** 10.1210/clinem/dgaf155

**Published:** 2025-04-11

**Authors:** Clemens Baumgartner, Matthäus Metz, Marianna Beghini, Lorenz Pfleger, Anna Tosin, Oliver Koldyka, Hannes Beiglböck, Paul Fellinger, Greisa Vila, Anton Luger, Alexandra Kautzky-Willer, Angelika Freudenthaler, Sabina Baumgartner-Parzer, Herbert Stangl, Martin Krssak, Fabrizia Carli, Patrizia Infelise, Amalia Gastaldelli, Thomas Scherer, Michael Krebs, Peter Wolf

**Affiliations:** Division of Endocrinology and Metabolism, Medical University of Vienna, Internal Medicine III, Waehringer Guertel 18-20, Vienna 1090, Austria; Division of Endocrinology and Metabolism, Medical University of Vienna, Internal Medicine III, Waehringer Guertel 18-20, Vienna 1090, Austria; Division of Endocrinology and Metabolism, Medical University of Vienna, Internal Medicine III, Waehringer Guertel 18-20, Vienna 1090, Austria; Division of Endocrinology and Metabolism, Medical University of Vienna, Internal Medicine III, Waehringer Guertel 18-20, Vienna 1090, Austria; Division of Endocrinology and Metabolism, Medical University of Vienna, Internal Medicine III, Waehringer Guertel 18-20, Vienna 1090, Austria; Division of Endocrinology and Metabolism, Medical University of Vienna, Internal Medicine III, Waehringer Guertel 18-20, Vienna 1090, Austria; Division of Endocrinology and Metabolism, Medical University of Vienna, Internal Medicine III, Waehringer Guertel 18-20, Vienna 1090, Austria; Division of Endocrinology and Metabolism, Medical University of Vienna, Internal Medicine III, Waehringer Guertel 18-20, Vienna 1090, Austria; Division of Endocrinology and Metabolism, Medical University of Vienna, Internal Medicine III, Waehringer Guertel 18-20, Vienna 1090, Austria; Division of Endocrinology and Metabolism, Medical University of Vienna, Internal Medicine III, Waehringer Guertel 18-20, Vienna 1090, Austria; Division of Endocrinology and Metabolism, Medical University of Vienna, Internal Medicine III, Waehringer Guertel 18-20, Vienna 1090, Austria; Division of Endocrinology and Metabolism, Medical University of Vienna, Internal Medicine III, Waehringer Guertel 18-20, Vienna 1090, Austria; Division of Endocrinology and Metabolism, Medical University of Vienna, Internal Medicine III, Waehringer Guertel 18-20, Vienna 1090, Austria; Center for Pathobiochemistry and Genetics, Medical University of Vienna, Institute of Medical Chemistry, Waehringer Straße 10, Vienna 1090, Austria; Division of Endocrinology and Metabolism, Medical University of Vienna, Internal Medicine III, Waehringer Guertel 18-20, Vienna 1090, Austria; Department of Biomedical Imaging and Image-Guided Therapy, Medical University of Vienna, High-Field MR Centre, Lazarettgasse 14, Vienna 1090, Austria; Cardiometabolic Risk Unit, Institute of Clinical Physiology, Consiglio Nazionale delle Ricerche (CNR), Via Giuseppe Moruzzi 1, Pisa 56124, Italy; Cardiometabolic Risk Unit, Institute of Clinical Physiology, Consiglio Nazionale delle Ricerche (CNR), Via Giuseppe Moruzzi 1, Pisa 56124, Italy; Cardiometabolic Risk Unit, Institute of Clinical Physiology, Consiglio Nazionale delle Ricerche (CNR), Via Giuseppe Moruzzi 1, Pisa 56124, Italy; Division of Endocrinology and Metabolism, Medical University of Vienna, Internal Medicine III, Waehringer Guertel 18-20, Vienna 1090, Austria; Division of Endocrinology and Metabolism, Medical University of Vienna, Internal Medicine III, Waehringer Guertel 18-20, Vienna 1090, Austria; Division of Endocrinology and Metabolism, Medical University of Vienna, Internal Medicine III, Waehringer Guertel 18-20, Vienna 1090, Austria

**Keywords:** growth hormone, acromegaly, hepatic lipid metabolism, very low-density lipoprotein, de novo lipogenesis

## Abstract

**Context:**

Growth hormone (GH) reduces intrahepatic lipids (IHL) according to investigations in healthy volunteers and patients with acromegaly, a disease characterized by long-term GH excess.

**Objective:**

This study investigated underlying antisteatotic pathways stimulated by short-term modulation of GH action.

**Methods:**

Ten healthy male volunteers (26 ± 5 years, body mass index [BMI] 23 ± 3.4 kg/m^2^) were assessed before and after 1 week of daily subcutaneous treatment with either GH or a GH-receptor antagonist in a crossover study (EK Nr.1395/2020; Eudra-CT:2020-000831-34). The assessments comprised the quantification of IHL and hepatic ATP synthesis via magnetic resonance spectroscopy, assessment of very low-density lipoprotein (VLDL) secretion by an intralipid infusion protocol, and measurement of de novo lipogenesis (DNL) using stable isotope tracer techniques. In comparison, effects of long-term GH excess on VLDL secretion were investigated in patients with active acromegaly (54 ± 5 years; BMI 29.3 ± 3.6 kg/m^2^; insulin-like growth factor I of 3.1 ± 1 × upper limit of normal).

**Results:**

GH treatment stimulated the secretion of VLDL-triglycerides by 26.1% (590.5 ± 282.3 mg/h vs 738.8 ± 424.9 mg/h, *P* = .035). Contrarily, mean DNL doubled after GH-receptor blockage without statistical significance (3.06 ± 1.95 vs 7.32 ± 8.43%, *P* = .107). Effects on hepatic ATP synthesis were not observed. Baseline hepatic VLDL secretion was comparable between volunteers and patients with acromegaly.

**Conclusion:**

GH modulates hepatic lipid turnover via an increase in hepatic triglyceride export and repressed GH action tends to foster DNL, which may be of assistance for the development of future therapeutic strategies against metabolic dysfunction–associated steatotic liver disease.

Hepatic steatosis represents one of the most common metabolic disorders in industrialized countries and is associated with obesity, type 2 diabetes, and the metabolic syndrome (1). Intrahepatic lipid content (IHL) rises whenever hepatic uptake and storage of lipids predominate over their disposal. This commonly occurs when overall lipid supply exceeds the storage capacity of white adipose tissue (WAT). Accompanying hyperinsulinemia and insulin resistance stimulate hepatic de novo lipogenesis (DNL) and peripheral lipolysis causing a fatty acid shift from WAT into ectopic sites like the liver (2-4). The resulting oversupply can be compensated by hepatic lipid export via the secretion of very low-density lipoprotein (VLDL) particles, which constitutes an important antisteatotic mechanism (5, 6). Increased intrahepatic lipid oxidation might represent another strategy to overcome an excessive influx of lipids (7, 8). In hepatic steatosis, however, both VLDL secretion and lipid oxidation are limited in their compensatory capacity, presumably due to saturation of both pathways (9, 10). Increased IHL, in turn, promotes both local and generalized insulin resistance, which makes steatosis the hepatic manifestation of a systemic, metabolic condition. Even though glucagon-like peptide 1 receptor agonists and thyroid hormone receptor agonists are considered to be beneficial in patients with metabolic dysfunction–associated steatohepatitis, available medical treatment beyond lifestyle modification remains scarce (11, 12).

Growth hormone (GH) is increasingly recognized in the context of IHL modulation (13, 14). As a major anabolic hormone during stress and famine, GH reduces protein breakdown and preserves stored glycogen by shifting overall catabolism to the mobilization and utilization of lipids. Effects on target tissues are either mediated directly by GH or indirectly via insulin-like growth factor I (IGF-I) secretion from the liver (15).

Consequences of long-term GH excess can be observed in acromegaly, a state of chronic GH hypersecretion. Patients with acromegaly exhibit a disease specific phenotype of lipid distribution with decreased visceral and subcutaneous WAT (16). Reductions in IHL have been repeatedly related to GH/IGF-I axis activity in individuals both without (17) and with acromegaly (18, 19), and a decrease in IHL was also reported in obese patients after 6 months of GH administration (20).

The biochemical pathways responsible for IHL reduction and the duration of GH alterations required for an antisteatotic effect are incompletely understood. Therefore, we studied the effects of short-term pharmacologic GH/IGF-I axis modification on hepatic lipid metabolism in humans using a crossover balanced within-subject study design. The results in healthy male volunteers were compared to those of patients with active acromegaly, serving as an in vivo model of long-term GH excess.

## Methods

The effects of short-term GH modulation on hepatic lipid metabolism were investigated in 10 healthy male volunteers (Cohort 1) in a single blinded, crossover balanced within-subject study design. Cohort 2 served as a model for endogenous, long-term GH excess and included patients with active acromegaly, in which hepatic lipid metabolism was assessed by cross-sectional analysis.

The study was approved by the ethics committee of the Medical University of Vienna (Ethics vote Nr. 1395/2020) and registered at the EudraCT database (2020-000831-34). Healthy volunteers were recruited based on advertisements. Patients were recruited at our center after diagnosis of active acromegaly. Written informed consent was obtained from all participants. Experiments were conducted at the Department of Internal Medicine III, Division of Endocrinology and Metabolism and the Centre for Pathobiochemistry and Genetics of the Medical University of Vienna, Austria. Study-related activities were conducted according to the declaration of Helsinki.

### Study Population

Inclusion criteria for Cohort 1 (healthy volunteers) comprised male sex, an age between 18 and 70 years, and serum triglycerides below 300 mg/dL. Participants of Cohort 2 had to be in the state of active acromegaly defined as diagnosed acromegaly without clinical remission and inherit serum triglycerides below 500 mg/dL.

Exclusion criteria for healthy volunteers were defined as a history of pancreatitis, simultaneous participation in another active clinical study, allergies against soy products, eggs, or peanuts, a glycated hemoglobin (HbA1c) > 6%, known liver disease (aspartate aminotransferase/alanine aminotransferase > upper limit of normal range [ULN]) or kidney disease (glomerular filtration rate [GFR] < 65 mL/min), consumption of alcoholic beverages during the last 48 hours before study-related activities, body mass index (BMI) > 30 kg/m2, tendency toward claustrophobia, and metal devices or other magnetic material in or on the body that would have been hazardous for magnetic resonance spectroscopy (MRS) investigation.

Exclusion criteria for Cohort 2 were a history of pancreatitis, simultaneous participation in another active clinical study, allergies against soy products, eggs, or peanuts, severe liver disease (liver function tests > 3 times ULN), and a GFR < 45 mL/min.

All participants were instructed to eat a balanced diet (55% carbohydrate, 20% fat, 15% protein), and to refrain from physical exercise and consumption of alcohol 2 days before study-related activities.

### Study Design

In vivo measurements of hepatic lipid metabolism were conducted after an overnight fast. At each study day, participants first underwent ^1^H/^31^P MRS for quantification of IHL and assessment of phosphor metabolite concentrations. Further study-related activities in the fasting state consisted of a clinical examination including the measurement of abdominal circumference, body composition analysis (BodPoD®), and blood sampling. Subsequently, hepatic VLDL-TG secretion was assessed via an Intralipid® infusion protocol (21).

#### Cohort 1

**Figure 1.**
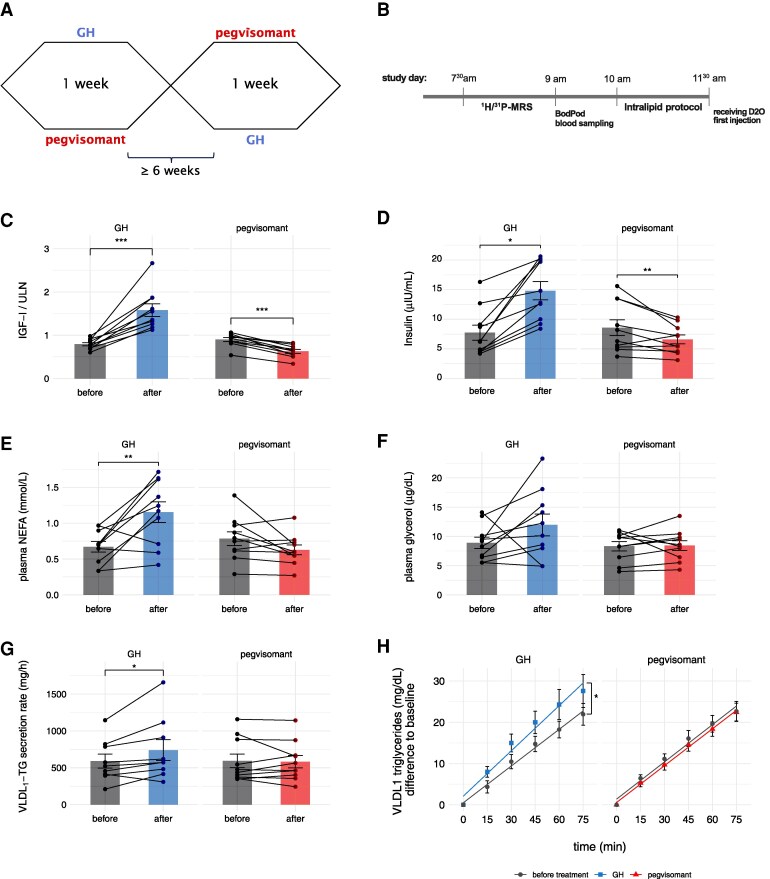
Short-term growth hormone excess stimulates hepatic triglyceride secretion of healthy males. A) Crossover study design. B) Time schedule of study days. C) Corrected insulin-like growth factor I concentrations (measured IGF-I divided by the individual upper limit of normal) of healthy males before and after each study week. D) Insulin concentrations before and after each study week. E) Concentrations of plasma non-esterified fatty acids before and after each study week. F) Concentrations of plasma glycerol before and after each study week. G) VLDL-TG secretion rates calculated from the linear increase of VLDL-TG during the intralipid infusion protocol adjusted for body weight (GH: n = 9, pegvisomant: n = 10). H) linear regressions of VLDL-TG accumulation over time. Data are presented as mean ± standard error of the mean (SEM). n = 10 for each study week if not stated otherwise. According to paired *t* test: *P < .05, ** *P* < .01, *** *P* < .001.

After completion of measurements, participants received either GH in the form of 2 mg GH (Genotropin®) or the GH-antagonist pegvisomant (Somavert®) (20 mg/day, loading dose of 40 mg at the first day) as daily, subcutaneous injections for 1 week (ie, 7 days), including the first study day. Patients received the first dose at our outpatient clinic and were trained for further self-injection of medication daily at 10 Pm. After 1 week of treatment, measurements of the first study day were repeated for intra-individual comparison. Following a washout period for at least 6 weeks, the study week was repeated with the respective other drug. Assignment to whether GH or pegvisomant would be administered in the first week was randomized using randomizer.org and the participant was blinded to the treatment. Details on the study design and the planning of study days are shown in Fig. 1A and 1B.

#### Cohort 2

Measurements of patients with acromegaly were conducted in the state of active disease similarly to Cohort 1. Study participation did not influence the decision of further therapeutic strategies, which were made only in respect of the individual clinical case. Before and after the study, clinical status, current medication, and laboratory results were examined by an endocrinologist.

### ^1^H/^31^P-Magnetic Resonance Spectroscopy

Phosphorus-31 and proton MRS (^31^P/^1^H MRS) were applied to study the energy metabolism as well as IHL, respectively. Data were acquired with a dual-tuned ^1^H/^31^P surface coil (Rapid Biomedical, Rimpar, Germany) on a 7T MR system (MAGNETOM, Siemens Healthineers, Erlangen, Germany). The subjects were positioned on the right side with the right liver lobe centered on top of the coil. To assess the concentrations of ^31^P metabolites a phantom replacement method was used with a 3-dimensional MRS imaging (3D MRSI) sequence (22). The ATP-synthase (ADP + Pi → ATP) rate k of the liver was assessed by ^31^P MRS saturation transfer (ST) as described (23). Furthermore, the ATP-flux was estimated by multiplying the ATP-synthase rate k with the Pi concentration (24). The IHL and a related unsaturation index were acquired according to the method of Gajdošik et al (25): a modified stimulated echo acquisition mode (STEAM) sequence with gradient-switching ultrashort echo time (GUSTEAU, TR = 5 second, TE = 6 msec, 8 measurements) was used to acquire ^1^H spectra of a single voxel (3 × 3 × 3 cm). This was measured both on the water resonance and with a delta frequency of −3.4 ppm (major lipid resonances) at 2 different positions within the liver tissue. Quantification results were corrected for both T1 and T2.

### Intralipid Infusion Test

For the in vivo assessment of VLDL triglyceride secretion rate, an intralipid infusion protocol was performed as described (21). The competition of VLDLs and chylomicrons for the same lipolytic pathway via lipoprotein lipase can be used to block VLDL degradation with a continuous infusion of chylomicron-like particles (Intralipid®, Fresenius Kabi Austria GmbH, Graz, Austria): For the procedure, intravenous cannulae were placed into the antecubital vein of both arms. After fasted blood sampling, administration of 20% Intralipid was started at 1 arm with a bolus of 0.1 g/kg bodyweight over 1 minute followed by an infusion of 0.1 g/kg bodyweight per hour (Supplementary Fig. S1) (26). Repeated blood samples were taken with EDTA tubes every 15 minutes over a period of 75 minutes and plasma was extracted by centrifugation at 2000*g* for 10 minutes. Subsequently, VLDL particles were isolated with density gradient ultracentrifugation and VLDL-triglycerides (VLDL-TG) were measured in each sample via a colorimetric assay (DiaSys Diagnostic Systems GmbH, Holzheim, Germany). The VLDL-TG secretion rate was calculated from the slope of the linear increase in triglyceride content (mg/dL) over time multiplied by 4% of body weight in kg (ie, plasma volume, see (27)) and by 60 to obtain the secretion rate per hour. More details on VLDL extraction are given in the supplementary material (26).

### Stable Isotope Tracer Techniques

During hepatic de novo lipogenesis (DNL) out of carbohydrates, hydrogen molecules are incorporated in newly synthesized palmitate. Using deuterated water (D_2_O) as a stable isotope tracer enables quantification of fatty acids derived from hepatic DNL via gas chromatography/tandem mass spectrometry (28). After the first study day of each round, participants of Cohort 1 were given 950 mL of 70% D2O (Cambridge Isotope Laboratories, USA) for oral intake in a prescribed regime for the whole study week (ie, 200 mL per day from day 1 to day 4, and 50 mL per day from day 5 to day 7). At day 4 and after the study week, blood samples were taken during the fasting state to determine D_2_O-labeled lipid species within VLDL particles. In collaboration with the Institute of Clinical Physiology, CNR, Pisa Italy, fasting DNL was quantified according to previously published protocols (28); the detailed methodology is further described within the supplementary material (26).

### General Blood Sampling and Biochemical Detection of Peripheral Lipolysis

General metabolic blood biomarkers and blood hormone concentrations were determined at the central laboratory of the General Hospital of Vienna (29) and included GH, IGF-I, insulin, C-peptide, glucose, serum triglycerides, cholesterol, high-density lipoprotein, and low-density lipoprotein. Further blood samples obtained during the study day (ie, in the fasting state and during the intralipid infusion protocol) were immediately stored on ice and centrifuged at 2000*g* for 10 minutes using a Megafuge ST4FR Plus centrifuge (Thermoscientific, Germany). Samples of EDTA plasma to determine the VLDL-TG secretion rate were processed immediately after the study day and VLDL-TG concentration was measured as described above. Serum samples to determine DNL via total VLDL fraction were either processed directly or stored at 4 °C for a maximum of 24 hours before ultracentrifugation. Plasma samples to determine non-esterified fatty acids (NEFA) (FUJIFILM Wako Chemicals, Neuss, Germany), glycerol (Sigma Aldrich, St. Louis, Missouri, USA), and ketone body concentrations (FUJIFILM Wako Chemicals) were immediately stored at −80 °C until analysis and concentrations were determined via colorimetric assays according to the manufacturer's instructions.

### Body Composition

Body composition analysis was conducted via body densitometry using a body composition tracking system (BodPod®, COSMED, USA) at every study day to accurately determine body mass, fat mass, and fat-free mass. Additional assessed anthropometric data included abdominal circumference and BMI.

### Statistical Analysis

For descriptive purposes, qualitative parameters are presented as count and percentage and quantitative parameters are presented as means ± SD. Testing for normal distribution was achieved by visual methods using histograms. In Cohort 1, continuous data were compared using a 2-sided paired *t* test. Intra-individual comparisons were depicted as plots showing mean, standard error of the mean (SEM), and lines as direct connections of individual data points. For the comparison of VLDL-TG secretion rates between baseline and treatment in Cohort 1, simple linear regression lines were used depicting baseline-adjusted VLDL-TG concentrations during the performed intralipid infusion (Fig. 1H). In both cohorts, associations between continuous variables were depicted by scatter plots with inserted regression lines and were described by Spearman's correlation coefficients, whereby Spearman's rho and level of significance were added to the plot. Unpaired T test was used for comparison of VLDL secretion between both cohorts. For testing, significance was determined by 2-sided α < .05. The R software (R Core Team. R: A Language and Environment for Statistical Computing. Vienna, Austria: R Foundation for Statistical Computing, 2020. https://www.R-project.org/) was used for all descriptive and analytical statistics.

## Results

### Short-Term Growth Hormone Excess Stimulates Hepatic Triglyceride Secretion of Healthy Males

Subject characteristics and laboratory parameters of Cohort 1 are summarized in Table 1. All 10 participants finished both weeks of treatment, the mean washout time between study weeks of 1 individual was 116 ± 67 days. Effective treatment is proven by significant changes in IGF-I after 1 week exposure to GH and pegvisomant (Fig. 1C). Effects on whole body metabolism were observed by significant changes of insulin (Fig. 1D) and C-peptide, which were discordant between both conditions. There were no significant changes in body composition (ie, BMI, fat mass, and fat-free mass). After GH, we observed a marked increase in plasma NEFA, as well as a concordant, but nonsignificant change in glycerol concentration as surrogates of adipose tissue lipolysis. No significant changes in plasma NEFA (Fig. 1E), ketone bodies, and glycerol concentrations (Fig. 1F) were observed after pegvisomant.

**Table 1.** Characteristics and biochemical parameters of healthy volunteers before and after GH/IGF-I axis modulation

	GH			Pegvisomant		
	Baseline	After treatment	*P* value	Baseline	After treatment	*P* value
Age, years	26.2 ± 5			26.1 ± 4.9		
BMI, kg/m^2^	23 ± 3.4			23 ± 3.4		
Abdominal circumference, cm	84.6 ± 10.2			85 ± 10.3		
Body weight, kg	79 ± 13.6	79.4 ± 13	.510	79.1 ± 13.1	78.9 ± 13.3	.327
Relative fat mass %	17.2 ± 7.3	16.8 ± 7.2	.079	17.5 ± 7.1	16.6 ± 7.6	.130
Relative fat-free mass %	82.8 ± 7.3	83.2 ± 7.2	.079	82.5 ± 7.1	83.4 ± 7.6	.130
GH, ng/mL	1.7 ± 2	2.5 ± 2.7	.460	4 ± 7.8	19.7 ± 6.5	.001
IGF-I, ng/mL	233 ± 44	449 ± 80	< .001	263 ± 62	184 ± 54	<.001
IGF-I divided by ULN	0.8 ± 0.1	1.6 ± 0.5	<.001	0.9 ± 0.2	0.6 ± 0.1	<.001
Glucose, mg/dL	85 ± 6	87 ± 10	.566	85 ± 6	84 ± 7	.425
Insulin, mcIU/mL	7.7 ± 4	14.8 ± 4.9	.001	8.6 ± 4.2	6.6 ± 2.4	.033
C-peptide, ng/mL	1.6 ± 0.4	2.4 ± 0.6	.004	1.7 ± 0.4	1.6 ± 0.4	.045
HOMA-IR	1.7 ± 0.9	3.2 ± 1.1	.004	1.8 ± 1	1.4 ± 0.6	.035
Triglycerides, mg/dL	97 ± 56	97 ± 25	.995	80 ± 28	96 ± 40	.130
Cholesterol, mg/dL	148 ± 23	138 ± 17	.019	152 ± 29	155 ± 25	.312
HDL, mg/dL	50 ± 8	48 ± 6	.274	51 ± 8	50 ± 9	.366
LDL, mg/dL	79 ± 18.8	70.3 ± 18.9	.051	84.7 ± 24.6	86.2 ± 21	.617
VLDL-TG secretion rate, mg/h	590.5 ± 282.3	738.8 ± 424.9	.035	593 ± 294.8	582.3 ± 267	.793
Plasma NEFA, mmol/L	0.7 ± 0.2	1.2 ± 0.5	.007	0.8 ± 0.3	0.6 ± 0.2	.092
Plasma glycerol, mcg/dL	8.9 ± 3.1	12 ± 5.9	.146	8.3 ± 2.5	8.4 ± 2.6	.868
Plasma ketone bodies, mg/dL	136.1 ± 126	518.8 ± 554.4	.057	156 ± 113.8	86.4 ± 46.2	.119
IHL (%)	3.1 ± 4.5	2.1 ± 1.6	.423	1 ± 0.4	1.5 ± 0.8	.053
UI	0.1 ± 0	0 ± 0	.139	0.1 ± 0	0.1 ± 0	.218
k_atp	0.2 ± 0.1	0.2 ± 0.1	.314	0.3 ± 0.1	0.3 ± 0.1	.946
atp_flux	0.4 ± 0.1	0.5 ± 0.2	.453	0.5 ± 0.2	0.5 ± 0.2	.354

Data are presented as mean ± SD, n = 10. Missing values are reported in the text and in Supplementary Table S1.

Abbreviations: BMI, body mass index; GH, growth hormone; HDL, high-density lipoprotein; HOMA-IR, homeostatic model assessment for insulin resistance; IGF-I, insulin-like growth factor I; IHL, intrahepatic lipid content; LDL, low-density lipoprotein; NEFA, non-esterified fatty acids; UI, unsaturation index; ULN, upper limit of normal; VLDL-TG, very low-density lipoprotein–triglycerides.

Data on VLDL-TG secretion were available for 9 subjects after GH treatment and for all subjects after pegvisomant. In 1 participant, VLDL-TG secretion during the GH week could only be sufficiently measured at baseline (328.4 mg/h) due to technical difficulties in the extraction procedure of VLDL fraction after 1 week of GH (Supplementary Fig. S2, Supplementary Table S1 (26)). Intra-individual comparison of VLDL-TG secretion showed a significant increase after 1 week of GH treatment (590.5 ± 282.3 mg/h vs 738.8 ± 424.9 mg/h, *P* = .035), which accounts for 26.1% compared to baseline. Conversely, VLDL-TG secretion did not differ before and after 1 week of pegvisomant (Fig. 1G and 1H). Correlations between parameters of hepatic lipid metabolism for Cohort 1 can be found in the supplementary material (Supplementary Table S3 (26)).

### Physiological IHL Content During Short-Term Growth Hormone Therapy

To determine IHL changes in response to short-term GH/IGF-I axis modulation, healthy participants underwent ^1^H-MRS at the beginning of each study day before and after treatment with GH or pegvisomant. Data were missing in 2 out of 10 participants before treatment with pegvisomant due to technical problems (Supplementary Table S1 (26)).

**Figure 2.**
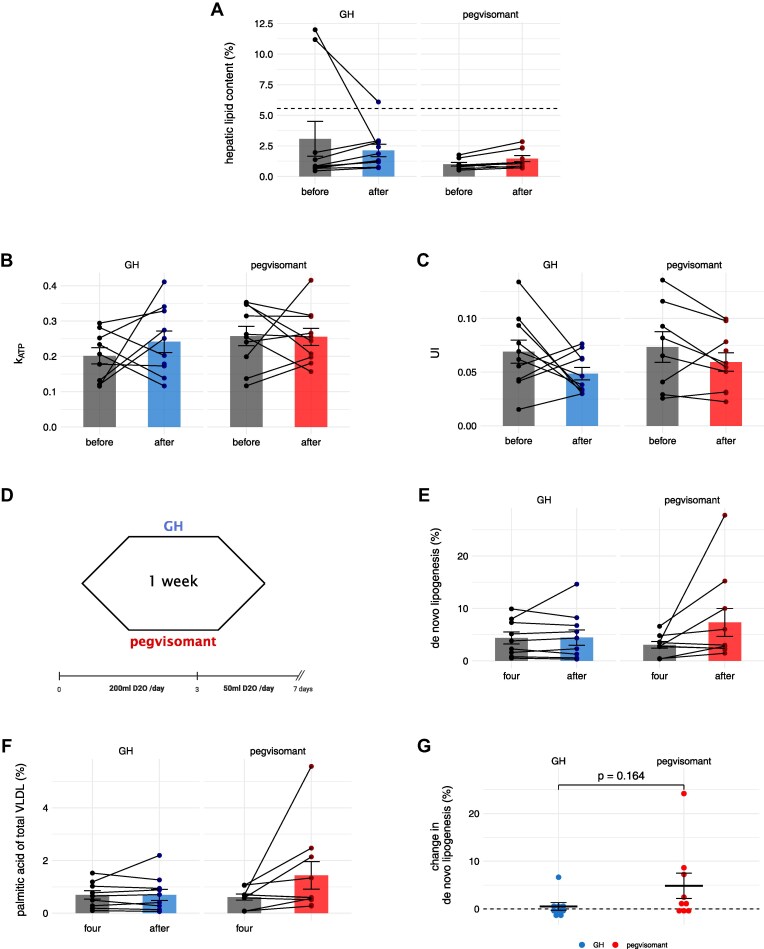
The impact of short-term growth hormone modulation on intrahepatic lipids, mitochondrial turnover, and de novo lipogenesis in healthy males. A) Intrahepatic lipid content before and after each study week (GH: n = 10, pegvisomant: n = 8). B) Hepatic k_ATP_ measured with ^31^P-MRS. C) Unsaturation index (UI) measured with ^31^P-MRS. D) Scheme of daily oral intake of deuterated water (D_2_O) during each study week. E) De novo lipogenesis before and after each study week. F) Palmitic acid concentrations of the total VLDL fraction before and after each study week. G) Compared changes of de novo lipogenesis between study weeks. Data are represented as mean ± standard error of the mean (SEM). n = 10 for each study week if not stated otherwise. According to paired *t* test: *P < .05, ** *P* < .01, *** *P* < .001.

Intra-individual baseline measurements of IHL were comparable between both weeks (*P* = .384). Among the participants, 2 of 10 subjects had high baseline IHL before GH treatment (11.2% and 12%), which showed a strong decrease after intervention. No significant changes in IHL were observed after both study weeks (see Fig. 2A). Within healthy volunteers, IHL correlated positively with VLDL-TG secretion rate (rho = 0.66, *P* < .001) as with other metabolic biomarkers, including BMI, relative fat mass, serum triglycerides, insulin, and homeostatic model assessment for insulin resistance (HOMA-IR). A negative correlation was observed with relative fat-free mass (Supplementary Table S3 (26)). There were neither correlations of IHL with plasma NEFA or glycerol nor between changes of IHL and plasma NEFA or glycerol.

### Short-Term GH/IGF-I Axis Modulation Does Not Alter Hepatic Unsaturation Index, Mitochondrial Activity, or Hepatic Phosphor Metabolite Concentrations

Data on hepatic phosphor metabolites assessed via ^31^P-MRS measurement are presented in Supplementary Table S2 (26). Hepatic k-ATP (Fig. 2B), hepatic unsaturation index (Fig. 2C), and ATP-flux remained unchanged after both treatments. Except a decrease of inorganic phosphate after 1 week of pegvisomant, no noticeable differences were observed in any other phosphor metabolites of healthy subjects. Regarding relations to hepatic lipid metabolism, noticeable correlations between VLDL-TG secretion rate, IHL, and phosphor metabolites are reported in the supplementary material (Supplementary Table S3 (26)).

### De Novo Lipogenesis During GH/IGF-I Axis Modulation in Healthy Humans

For intra-individual comparison of DNL between both study weeks, participants received D_2_O during each study week for daily oral intake according to a standardized protocol (Fig. 2D). Consumed D_2_O served as stable isotope tracer, since deuterium is incorporated in newly synthesized fatty acids via hepatic DNL, whereby a steady state of deuterium saturation was presumed from the fourth study day on. Hereby labeled lipids were quantified within the overall VLDL fraction of blood plasma by gas chromatography/mass spectrometry (28) at day 4 (ie, the day after the third injection of GH or pegvisomant) and after each study week.

Neither DNL (from 4.38 ± 3.41 to 4.44 ± 4.57%, *P* = .538) nor relative content of palmitic acid within total VLDL (from 0.69 ± 0.48 to 0.69 ± 0.67%, *P* = .577) differed between day 4 and after 1 week of GH. However, both DNL (from 3.06 ± 1.95 to 7.32 ± 8.43%, *P* = .107) and VLDL-palmitic acid content (from 0.61 ± 0.35 to 1.43 ± 1.65%, *P* = 0.114) doubled during 3 days of active pegvisomant treatment, even though a wide variation was observed (Fig. 2E and 2F) and the change was not statistically significant. Comparison of absolute changes between 3 days of GH (0.52 ± 2.42%) and pegvisomant (4.83 ± 7.99%) did not reach statistical significance (*P* = .164); see Fig. 2G. There were no relevant correlations of DNL with IHL, VLDL-TG secretion, or IGF-I concentration (Supplementary Table S4 (26)).

### Hepatic Lipid Metabolism in Patients With Active Acromegaly

For the investigation during long-term GH excess, VLDL-TG secretion rate, IHL, biomarkers of peripheral lipolysis and hepatic phosphor metabolites were assessed in 6 patients (4 female, 2 male) with active acromegaly (Cohort 2). Of those, 3 patients presented with newly diagnosed acromegaly and 3 were not in remission after transsphenoidal macroadenoma-resection. No patient was on pharmacological treatment of acromegaly before study inclusion.

**Figure 3.**
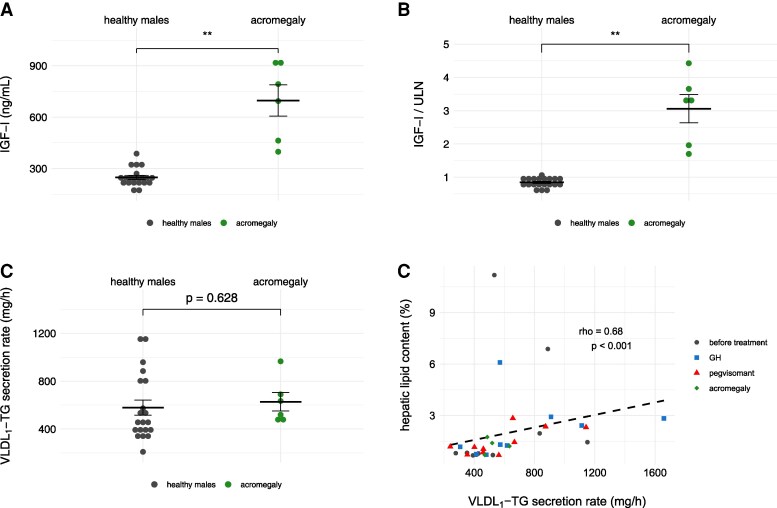
Relating hepatic lipid metabolism of healthy volunteers to an unmatched cohort of patients with acromegaly. A) Insulin-like growth factor I (IGF-I) concentrations of healthy volunteers before study weeks compared to patients with acromegaly. B) Corrected IGF-I concentrations (measured IGF-I divided by the individual upper limit of normal). C) VLDL-TG secretion rates of healthy volunteers before study weeks compared to patients with acromegaly. D) Correlation analysis between hepatic lipid content and VLDL-TG secretion rates of all observations and participants (patients with acromegaly: n = 4). In healthy volunteers, the mean value of both baseline measurements (ie, measurements before both study weeks) was used for analysis to overcome significance due to repeated measurements during equal conditions. Data are represented as mean ± standard error of the mean (SEM). Observations of healthy volunteers before respective study weeks: n = 20, after Genotropin n = 10, and after pegvisomant n = 10, acromegaly, n = 6 if not stated otherwise. According to unpaired *t* test: ** *P* < .01. *rho* indicates Spearman correlation coefficient.

Baseline characteristics and blood laboratory parameters are listed in Table 2. When adjusting IGF-I concentrations for the individual upper limit of normal, concentrations markedly differed between baseline IGF-I levels of patients with acromegaly and healthy volunteers (3.1 ± 1 vs 0.8 ± 0.1, *P* = .003; Fig. 3B). The average VLDL-TG secretion rate of acromegaly patients was 627.7 ± 186.7 mg/h. As seen in Fig. 3C, hepatic triglyceride export in patients with acromegaly did not significantly differ compared to our unmatched group of healthy males before GH-axis modulation (*P* = .628) and IHL of patients was low and comparable to previous studies (18, 19). The correlation between IHL and VLDL-TG secretion was observed in both acromegaly and healthy volunteers during any condition of GH-axis modulation or baseline (Fig. 3D).

**Table 2.** Demographic data and markers of hepatic lipid metabolism in patients with active acromegaly

N = 6		
Age	53.5 ± 5.21	6
Sex:		6
m	2 (33.3%)	
w	4 (66.7%)	
BMI	29.3 ± 3.59	6
Macroadenoma	6 (100%)	
Previous resection	3 (50%)	
Relative fat mass %	30.0 ± 9.12	6
Relative fat-free mass %	70.0 ± 9.12	6
GH, ng/mL	18.3 ± 12.0	6
IGF-I, ng/mL	697 ± 224	6
IGF-I divided by ULN	3.1 ± 1	6
Glucose, mg/dL	101 ± 11.4	6
Insulin, mcIU/mL	17.6 ± 13.1	6
C-peptide, ng/mL	3.62 ± 1.80	6
HOMA-IR	4.58 ± 4.06	6
HbA1c %	5.66 ± 0.26	5
Triglycerides, mg/dL	71.5 ± 18.4	6
Cholesterol, mg/dL	188 ± 39.5	6
HDL, mg/dL	65.5 ± 20.2	6
LDL, mg/dL	109 ± 33.3	6
VLDL-TG secretion rate, mg/h	627.7 ± 186.7	6
Plasma NEFA, mmol/L	0.50 ± 0.12	5
Plasma glycerol, mcg/dL	7.11 ± 1.49	5
Plasma ketone bodies, mg/dL	101 ± 72.0	5
IHL (%)	1.26 ± 0.43	4
UI	0.09 ± 0.09	4
k_atp	0.22 ± 0.06	4
atp_flux	0.41 ± 0.09	4

Data are presented as mean ± SD, n = 6. Numbers in the right column indicate the availability of data points per variable. IGF-I divided by ULN represents relative IGF-I concentrations reported as the ratio between measured IGF-I and the individual IGF-I-upper limit of normal. For healthy subjects, relative IGF-I concentrations are given in the text.

Abbreviations: BMI, body mass index; GH, growth hormone; HbA1c, hemoglobin A1c; HDL, high-density lipoprotein; HOMA-IR, homeostatic model assessment for insulin resistance; IGF-I, insulin-like growth factor I; IHL, intrahepatic lipid content; LDL, low-density lipoprotein; NEFA, non-esterified fatty acids; UI, unsaturation index; VLDL-TG, very low density-lipoprotein–triglycerides.

## Discussion

Our findings reveal in vivo effects of short-term GH/IGF-I axis modulation on hepatic lipid metabolism. GH excess stimulates hepatic VLDL-TG secretion. There was no significant increase of DNL after GH-receptor blockade, even though we observed a numerical change in DNL after pegvisomant. Whereas no significant change in IHL after short-term axis modulation was observed, long-term increased GH/IGF-I axis activity, as in patients with acromegaly, may cause low IHL content due to continuously stimulated hepatic lipid turnover. Conversely, the nonsignificant increase in DNL following GH-receptor antagonist treatment may indicate a worsening of hepatic lipid metabolism after blockage of GH/IGF-I axis activity.

### Hepatic Triglyceride Export During Short-Term GH-Axis Modulation

Our data reveal an acute effect of an increase in GH/IGF-I activity on VLDL kinetics. We consider an increase in VLDL-TG secretion of approximately 25% after GH treatment efficient to remove intrahepatic lipids and to represent a marker for increased intrahepatic lipid turnover (6).

In previous studies, a 3-month period of GH replacement in patients with GH deficiency resulted in a significant increase of VLDL-ApoB100 secretion assessed by stable isotope turnover techniques (30). In contrast, estimated VLDL secretion calculated from plasma disappearance rates did not show any changes in VLDL triglyceride kinetics after 8 days of GH administration (31). Methodological discrepancies between these studies hamper the sufficient interpretation of VLDL turnover, since a single administration of ex vivo labeled [1-14C] triolein VLDL-triglycerides used by Krag et al only allows for indirect estimation of VLDL secretion calculated from plasma disappearance rate, whereby Christ et al used a stable isotope turnover technique with a primed constant [1-13C] leucin infusion, enabling direct measurement of enriched VLDL particles.

### Hepatic Lipid Oxidation According to ^31^P-MRS Measurements

Hepatic ATP synthesis rate did not differ between short-term GH/IGF-I axis activation and suppression. In accordance, plasma ketone bodies, acting as a surrogate marker for hepatic lipid oxidation (32), remained unaltered in response to both treatments. These findings contrast with former investigations of mitochondrial activity in active acromegaly, in which patients exhibited significantly higher ATP-flux and ATP synthesis rates measured via ^31^P-MRS compared to matched controls (18). Replacement of GH was also followed by increased hepatic lipid oxidation in patients with GH deficiency (33). Thus, we assume that the increase in hepatic lipid oxidation monitored by ATP synthesis rate might only be of relevance following longer exposure to alterations in GH/IGF-I axis activity.

### Assessment of De Novo Lipogenesis With Stable Isotope Tracer Techniques

Rodent models reported an attenuation of hepatic DNL as a response to modulations in GH signaling (34): liver-specific GH-receptor knockdown increased DNL independently of the known regulatory pathways via insulin and was followed by the development of hepatosteatosis (35). An in vitro model of human HepG2 hepatocytes also showed a GH dependent suppression on DNL (36). Notably, human studies reported increased DNL in participants with high IHL (37). However, in vivo studies examining physiological GH action on DNL in healthy humans are missing.

In our study, a numerical increase in DNL with a wide variation and without statistical significance was observed after pegvisomant treatment, wherefore inhibitory effects of GH can only be suggested. The missing direct impact of GH after 1 week of treatment may be explained by an already suppressed liver DNL at baseline in healthy insulin sensitive volunteers.

Stable isotope tracer techniques are a reliable method to assess hepatic DNL during in vivo human study designs (38). According to recent reports, prolonged saturation periods were followed by higher rates of DNL compared to analyses 24 hours after D_2_O intake (28), which is why we decided for a 1-week exposure in our study protocol.

### Hepatic Triglyceride Secretion in Active Acromegaly

Acromegaly represents an in vivo model of long-term GH excess and is a unique condition of reduced IHL in the presence of insulin resistance (18, 19, 39). Previous studies reported an increase in hepatic mitochondrial activity, which may partially explain the observed reduction in IHL (18). We characterized hepatic VLDL-TG secretion during active disease. Considering the correlation between IHL and VLDL secretion, the observed low VLDL secretion in study participants with low baseline IHL seems reasonable. However, short-term GH excess as stimulus for enhanced hepatic triglyceride secretion observed in the healthy cohort substantiates the GH guided promotion of hepatic lipid turnover. We therefore speculate that preserved potency of triglyceride secretion following long-term GH excess may contribute to the low IHL observed within acromegaly.

## Limitations

Only male participants were included for investigations in healthy subjects. Because of the antisteatotic effects of estrogen (40) and the higher energy storage capacity of women (41), we refrained from investigating lipid metabolism in healthy female participants during this first analysis. Experiments in rodents reported higher VLDL secretion rates in female compared to male mice (42) which would further have biased results regarding the main outcome parameter of our study. Given the exploratory analysis in acromegaly, we included both female and male patients since former observations did not reveal any sex differences in states of long-term GH excess (18, 19). Dosing of GH and pegvisomant was chosen according to previously published protocols, in which similar doses efficiently stimulated (31) and repressed (43) GH after a treatment period of approximately 1 week.

Our cohort of healthy volunteers revealed 2 participants with initial IHL above the steatosis cutoff of 5.56% before the GH study week. Patients with steatosis are considered to respond to GH action via a decrease in IHL (17, 20), wherefore future studies will need to separately evaluate the dynamics of VLDL secretion of these patients.

According to logistic considerations, DNL was assessed only in healthy subjects: the data of patients with acromegaly shall primarily give a descriptive overview of VLDL kinetics within this rare disease model. Therefore, unmatched cohorts of healthy males and patients with acromegaly were also compared in a descriptive manner. Similar secretion rates in healthy males and acromegaly, however, were considered to reasonably depict VLDL secretion to be independent of long-term GH alterations.

## Conclusion and Future Perspectives

In concluding, short-term growth hormone excess enhances hepatic lipid turnover via an increase in hepatic triglyceride export. Depicted by a numerical change without statistical significance, medically induced GH blockade may tend to increase hepatic DNL, although this cannot be fully stated regarding the observed population. Our findings underline the importance of short-term GH/IGF-I modulation for hepatic lipid metabolism, provide insights into GH-mediated antisteatotic mechanisms and may be of assistance for the development of future therapeutic strategies against metabolic dysfunction–associated steatotic liver disease.

## Data Availability

Data will be made available upon reasonable request.

## Abbreviations

BMI: body mass index
D_2_O: deuterated water
DNL: de novo lipogenesis
GH: growth hormone
IGF-I: insulin-like growth factor I
IHL: intrahepatic lipids
MRS: magnetic resonance spectroscopy
NEFA: non-esterified fatty acids
ULN: upper limit of normal
VLDL: very low-density lipoprotein
WAT: white adipose tissue
